# USP8 inhibition regulates autophagy flux and controls *Salmonella* infection

**DOI:** 10.3389/fcimb.2023.1070271

**Published:** 2023-03-21

**Authors:** John Santelices, Mark Ou, Gustavo H. B. Maegawa, Kamil Hercik, Mariola J. Edelmann

**Affiliations:** ^1^ Department of Microbiology and Cell Science, Institute of Food and Agricultural Sciences, University of Florida, Gainesville, FL, United States; ^2^ Department of Pediatrics and Genetics, Columbia University Irving Medical Center, Vagelos Physicians and Surgeons College of Medicine, New York, NY, United States; ^3^ Institute of Organic Chemistry and Biochemistry, Academy of Sciences of the Czech Republic, Prague, Czechia; ^4^ Department of Molecular Pathology and Biology, Faculty of Military Health Sciences, University of Defense, Hradec Kralove, Czechia

**Keywords:** deubiquitinating enzymes, deubiquitinases, *Salmonella*, USP8, autophagy, chemical proteomics

## Abstract

**Introduction:**

Ubiquitination is an important protein modification that regulates various essential cellular processes, including the functions of innate immune cells. Deubiquitinases are enzymes responsible for removing ubiquitin modification from substrates, and the regulation of deubiquitinases in macrophages during infection with *Salmonella* Typhimurium and *Yersinia enterocolitica* remains unknown.

**Methods:**

To identify deubiquitinases regulated in human macrophages during bacterial infection, an activity-based proteomics screen was conducted. The effects of pharmacological inhibition of the identified deubiquitinase, USP8, were examined, including its impact on bacterial survival within macrophages and its role in autophagy regulation during *Salmonella* infection.

**Results:**

Several deubiquiitnases were differentially regulated in infected macrophages. One of the deubiquitinases identified was USP8, which was downregulated upon *Salmonella* infection. Inhibition of USP8 was associated with a decrease in bacterial survival within macrophages, and it was found to play a distinct role in regulating autophagy during *Salmonella* infection. The inhibition of USP8 led to the downregulation of the p62 autophagy adaptor.

**Discussion:**

The findings of this study suggest a novel role of USP8 in regulating autophagy flux, which restricts intracellular bacteria, particularly during *Salmonella* infection.

## 1 Introduction

Pathogenic *salmonellae* are one of the leading causes of enteric infections in the USA. Unfortunately, infectious diarrhoeal diseases are the second leading cause of death in children under five. The current control measures to prevent infections caused by *Salmonella* (NTS) are not optimal, and the emergence of multi-drug resistant (MDR) strains indicates that antimicrobial procedures need improvement (Coward et al., 2014). Because of these issues, new antimicrobials are urgently required, and WHO set the development of novel therapeutics against these and other pathogens as one of its top priorities. A better understanding of the molecular mechanisms involved in the host responses to Gram-negative pathogens can unravel new targets for drug design. The ubiquitin system constitutes one of the possible drug targets. Ubiquitin-guided proteolysis is one the most effective means of controlling the half-lives of proteins and regulating their function. However, ubiquitination has multiple functions in cells beyond its role in the targeted degradation of proteins (Swatek and Komander, 2016). Because of these reasons, targeting UPS family proteins has been recognized as offering significant new drug discovery opportunities across various therapeutic areas (Edelmann et al., 2011; Farshi et al., 2015; Lopez-Castejon and Edelmann, 2016; Lamberto et al., 2017; Weisberg et al., 2017). The ubiquitin system is a vital biological pathway involving more than 1,000 elements (Edelmann et al., 2011). The ability of the ubiquitination machinery to selectively target substrates is mediated by the specificity of ubiquitin ligation, catalyzed by E2 and E3 enzymes, and deconjugation, catalyzed by DUBs (Edelmann et al., 2011). Deubiquitinases (DUBs) are a family of proteases that reverse the ubiquitination process by cleaving ubiquitin from substrate proteins or reducing ubiquitin chains to their constituent monomers (Reyes-Turcu et al., 2009). Almost 100 genes encode deubiquitinases in humans, and these enzymes can be classified into two classes; cysteine proteases and metalloproteases. DUBs are categorized into seven deubiquitinase sub-groups, including the ubiquitin-specific proteases (USPs), the ovarian tumor proteases (OTUs), JAB1/MPN/MOV34 metalloproteases (JAMMs), the ubiquitin C-terminal hydrolases (UCHs), the Josephins, the motif interacting with ubiquitin (MIU)-containing novel deubiquitinase family (MINDYs), and ZUP1 (Komander et al., 2009). The ubiquitin-proteasome system plays a vital role in the host response to Gram-negative infections (Lopez-Castejon and Edelmann, 2016), and previous studies have shown that bacterial pathogens can interfere with the deubiquitinase-mediated removal of ubiquitin modifications (Kummari et al., 2015; Alugubelly et al., 2016; Hui et al., 2018). Concurrently, the host also regulates the activity and level of deubiquitinases to its advantage.

We hypothesized that inhibiting deubiquitinases in the host can promote bacterial clearance, which could open potentially novel targets for host-directed therapeutics against Gram-negative infections. Using a chemical proteomics approach, we compared the DUB activity profile in *Salmonella Typhimurium*-infected macrophages to *Yersinia enterocolitica-*infected cells, where *Y. enterocolitica* is another Gram-negative bacterium that uses Type Three Secretion System (TTSS). Since the TTSS effectors and their respective processes differ between these bacteria, we expected different deubiquitinase activity profiles. As proof of principle, we have validated one of the enzymes significantly downregulated during infection with *Salmonella*, USP8. We have furthermore determined that this deubiquitinase leads to the downregulation of p62 autophagosome cargo protein and regulates intracellular survival of *Salmonella*.

## 2 Results

### 2.1 Monitoring activity of deubiquitinases in macrophages during infection with *Salmonella* Typhimurium and *Yersinia enterocolitica*

**Figure 1:**
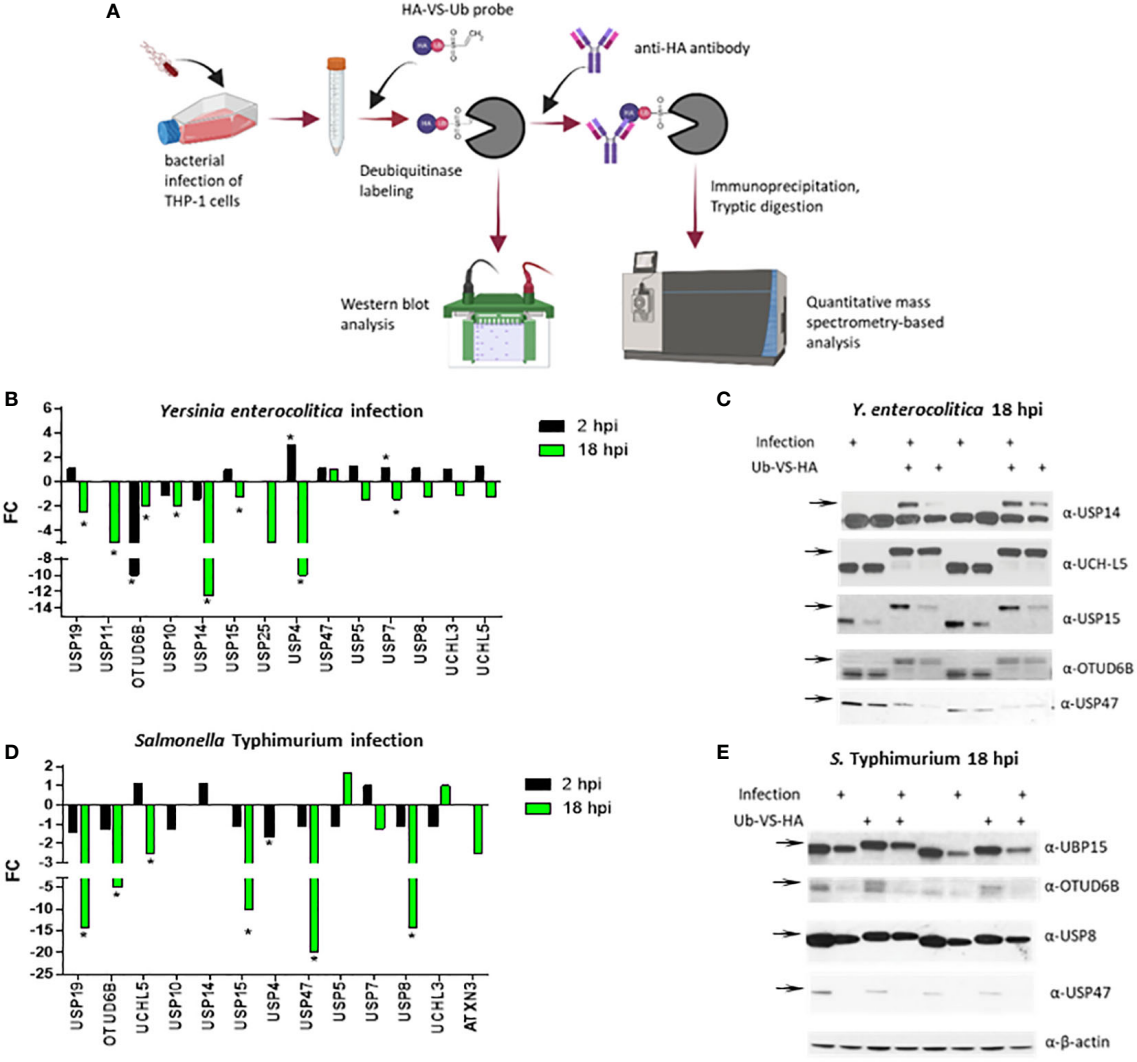
Activity-based labeling of DUBs in *Y. enterocolitica* or *S.* Typhimurium infection of human macrophages. Combining active site-directed probes and quantitative proteomics to identify changes in the DUB activity and (or) abundance in THP-1 cells **(A)** infected with *Y. enterocolitica* **(B, C)** or *S.* Typhimurium **(D, E)** or for 2 or 18 hours. **(A)** An overall strategy to label deubiquitinases in infected macrophages. Cells were left uninfected or infected with the bacteria indicated below and collected after each time point of infection. The cells were lysed to extract proteins, which was followed by the labeling of active deubiquitinases with ubiquitin-specific active site-directed probes (Ub-VS-HA). The probe-containing deubiquitinases were purified by anti-HA immunoprecipitation, and either visualized by using SDS-PAGE and western blotting or subjected to proteomics analysis to quantify the probe-bound deubiquitinases. The number of independent replicates for this experiment was 3 (n=3). **(B, D)**. The quantification of active DUBs in *Y. enterocolitica* or *S.* Typhimurium infection using proteomics. After mass spectrometric analysis of immunopurified deubiqutinases, the fold change differences between infected and uninfected cells were calculated from each protein’s total weighted spectral counts. The statistical analysis was done using Fisher’s test. The number of independent replicates for this experiment was 3 (n=3). **(C, E)**. The confirmation of protein abundance and activity of deubiquitinases by western blotting of the samples containing Ub-VS-HA probe labeling. The experiment described above was repeated, but instead of immunopurification, crude lysates (control samples containing no Ub-VS-HA labeling), and probe-reacted deubiquitinases (shown by increased molecular weight upon Ub-VS-HA binding) were visualized by using western blotting. The number of independent replicates for this experiment was 3 (n=3). Biological duplicates are shown as analyzed by western blot. P-values were indicated: * p≤ 0.05.

Activity-based proteomics enables monitoring the activity and expression of multiple enzymes concurrently. A chemical proteomics-based approach based on activity-based probes (ABPs) and combined with mass spectrometry-based proteomics can be used to identify and quantify DUBs in the cell lysates. These ABPs are often inhibitors that form covalent bonds with the enzyme of interest, such as deubiquitinases. Deubiquitinase-specific ABPs consist of a ubiquitin molecule fused to the Cysteine-reactive electrophilic group and can include a tag allowing for the purification and detection of probe-reactive enzymes (Borodovsky et al., 2001; Kessler, 2006). The electrophilic group reactive with the Cysteine residue or a warhead are propargylamide (PA) (Ekkebus et al., 2013; Sommer et al., 2013), vinyl methyl ester (VME) (Borodovsky et al., 2002), vinyl methyl sulfone (VS) (Borodovsky et al., 2001), chloroethylamine (Borodovsky et al., 2002), or bromoethylamine (Borodovsky et al., 2002). In this study, we used a VS-based ubiquitin probe (HA-Ub-VS) (Borodovsky et al., 2002), HA-Ub-VS, to monitor the abundance and activity of DUBs in the macrophages infected with two different Gram-negative bacteria. Human THP-1 macrophages were infected with *Salmonella* Typhimurium or *Yersinia enterocolitica* for 2 and 18 hours, followed by cell lysis and HA-Ub-VS probe labeling of deubiquitinases (**Figure 1A**). We used chemical proteomics approach, where deubiquitinases were reacted with active site-directed probes. Such probe-tagged enzymes were immunopurified using anti-HA antibodies and subjected to quantitative mass spectrometry analysis. Alternatively, following the probe labeling, the deubiquitinates were analyzed by western blotting. Based on the mass spectrometric analysis, the THP-1 macrophages infected with *Y. enterocolitica* for 2 hours post-infection (hpi) had an increased level of USP4 and USP7 but decreased level of OTUD6B (**Figure 1B**). At 18 hpi, the THP-1 macrophages had a decreased level of USP19, USP11, USP10, OTUD6B, USP15, USP4, and USP7 (**Figure 1B**). The western blotting analysis of the probe-reacted deubiquitinases was used as a validation, where the level of USP14, USP15, OTUD6B and USP47 was diminished during infection with *Y. enterocolitica* at 18 hpi (**Figure 1C**). The western blotting data indicate that in some cases the protein level was not significantly affected by the infection, but the activity level of the same enzymes (based on the Ub-VS-HA probe binding that increases the molecular weight of the active deubiquitinases) was diminished (**Figure 1C**), for example for USP14. Moreover, we used mass spectrometry analysis to evaluate the active deubiquitinase levels in the *S.* Typhimurium-infected macrophages. This infection did not lead to significant changes in deubiquitinase levels/activity at 2 hpi, apart from the downregulation of USP4 (**Figure 1D**). Based on the chemical mass spectrometry analysis, several deubiquitinases were downregulated by *S.* Typhimurium infection at 18 hpi, including USP19, OTUD6B, UCH-L5, USP15, USP47, and USP8 while other deubiquitinases remained unchanged (**Figure 1D**). The western blotting analysis (**Figure 1E**), confirmed the downregulation of protein levels of UBP15, OTU6B, USP8 and USP47 during the *S.* Typhimurium infection.

### 2.2 USP8 is downregulated during *S.* Typhimurium infection

**Figure 2:**
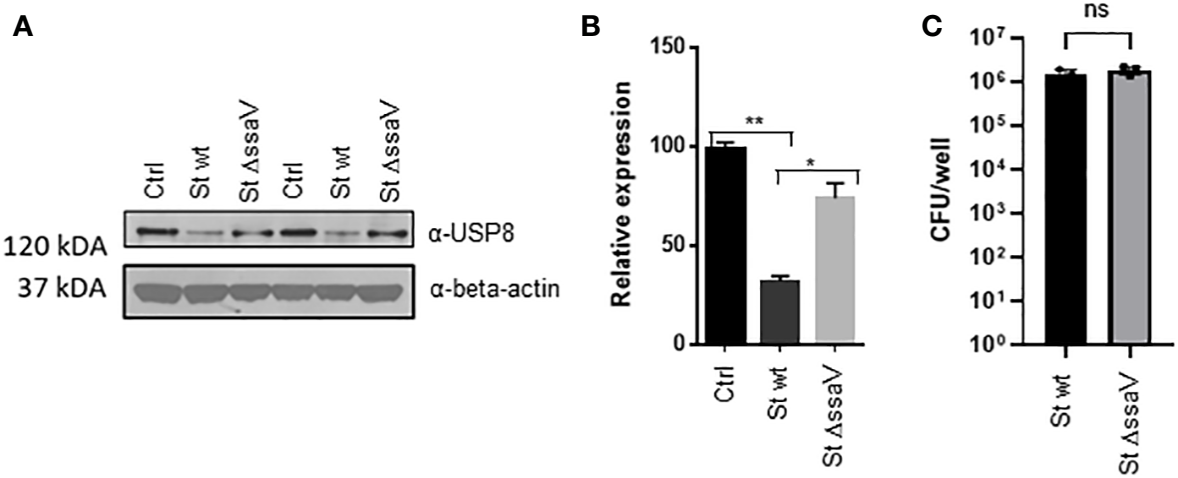
*S.* Typhimurium downregulates USP8 expression in macrophages and depends on the SPI-2 expression. **(A, B)**. THP-1 cells were infected with *S.* Typhimurium wild type or Δ*ssaV* lacking SPI-2 for 24 hours. Each sample was lysed, followed by western blotting to detect USP8 or loading control (β-actin; **A**). The abundance of USP8 was normalized to the β-actin as measured by ImageJ. The results of USP8 abundance are shown in a graphical form, where the statistical significance was determined by 1-way ANOVA **(B)**. The number of independent replicates for this experiment was 2 (n=2). **(C)**. Intracellular bacteria in THP-1 macrophages infected for 24 hours with *S.* Typhimurium wt compared to Δ*ssaV* mutant. The intracellular counts were obtained by using a gentamicin protection assay. The number of independent replicates for this experiment was 3 (n=3). P-values were indicated as follows: * p≤ 0.05; ** p ≤ 0.01; ns, not significant.

Mass-spectrometry-based quantification of active deubiquitinases established that USP8 was downregulated during *Salmonella* infection but not affected by *Y. enterocolitica* infection. To verify the down-regulation/inhibition of this deubiquitinase further, THP-1 cells were infected for an extended time of 24 hours with wild-type *S.* Typhimurium or a mutant lacking SsaV gene, which cannot express *Salmonella* Pathogenicity Island-2 (SPI-2) effectors. While the level of USP8 in THP-1 macrophages infected with wild-type *S.* Typhimurium was diminished, the infection with Δ*ssaV* did not lead to the downregulation of USP8 (**Figures 2A, B**), although the levels of intracellular bacteria were comparable for both strains (**Figure 2C**). This phenomenon might not be cell type-specific because similar results were observed in epithelial HeLa cells expressing FLAG-USP8, where USP8 was downregulated in infected HeLa cells at 24 hpi, but not at 2 hpi (**Figure S1**).

### 2.3 Effect of USP8 inhibition on cell viability and cytotoxicity

**Figure 3:**
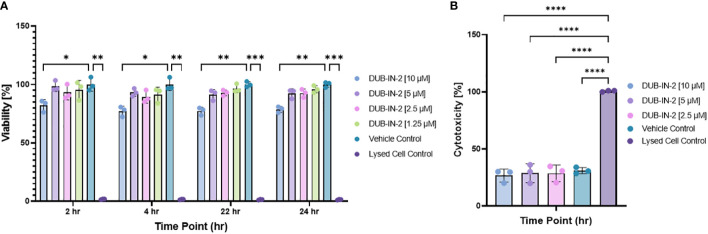
**(A)** Effect of USP8 inhibition on cell viability during infection with *Salmonella*. MultiTox-Fluor Multiplex Cytotoxicity assays were performed using four increasing concentrations of DUBs-IN2 inhibitor in THP-1 macrophages as indicated on the figures. Cells were treated with either vehicle control or DUBs-IN2 for either a 2- or 24-hour period, after which the assay buffers were added and incubated for 30 minutes before fluorescence was read on the Cytation3 Imaging Reader. The viability was calculated relative to the Vehicle control (100%), and the significance was established by One-way ANOVA test followed by multiple testing corrections, where the significance shows the differences between the vehicle control and each separate treatment The number of independent replicates for this experiment was 3 (n=3). **(B)**. Effect of USP8 inhibition on cell cytotoxicity during infection with *Salmonella*. Cells were subjected to the infection and DUBs-IN2 inhibitor treatment as indicated above. The cytotoxicity was measured by using MultiTox-Fluor Multiplex Cytotoxicity assay at 24 hours post-infection and shown as % where Lysed Cell Control represents 100% cytotoxicity. The significance was established by One-way ANOVA test followed by multiple testing corrections, where the significance shows the differences between each treatment. The number of independent replicates for this experiment was 3 (n=3). P-values were indicated as follows: * p≤ 0.05; ** p ≤ 0.01; *** p≤ 0.001; **** p ≤ 0.0001.

The effects of pharmacological inhibition of USP8 were tested in cells infected or not with *S.* Typhimurium. In this experiment, membrane-permeant DUBs-IN2 inhibitor was used as a highly selective inhibitor of USP8, which reaches a half maximal inhibitory concentration (IC_50_) of 0.28 μM (Colombo et al., 2010). Before measuring the effect of USP8 inhibition on bacterial infection, we first established the cell viability and cytotoxicity upon *Salmonella* infection in the presence or absence of this compound. THP-1 macrophages were treated with various amounts of the DUBs-IN2 inhibitor and subjected to infection with *Salmonella*, where the viability and cytotoxicity were measured at 2, 4, 22 and 24 hours post-infection (hpi). The THP-1 macrophages exposed to DUBs-IN2 USP8 inhibitor had decreased in viability only at the compound’s highest [10 µM] concentration, and the change in cytotoxicity was not statistically significant (**Figures 3A, B**). To ensure that these effects are not one-cell line-specific, we also tested viability in RAW 264.7 murine macrophages and HeLa cells, showing that the cell viability was not significantly affected by the inhibitor in these cells for any of the time points (**Figure S2**).

### 2.4 USP8 inhibition leads to decreased bacterial survival in macrophages

**Figure 4:**
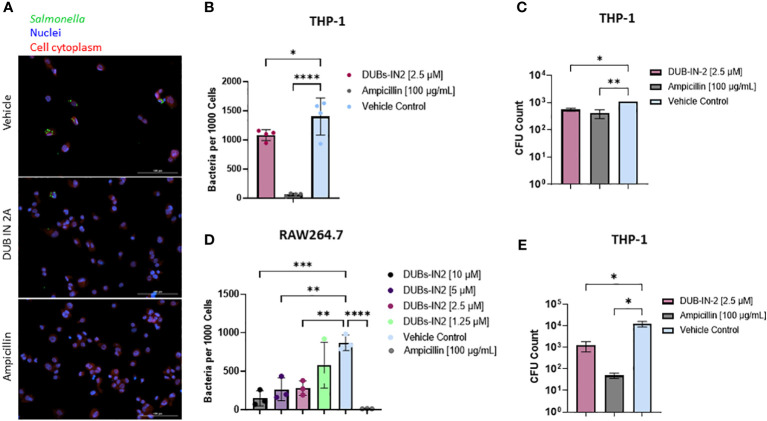
Impact of USP8 inhibition on intracellular *Salmonella* growth. **(A-C)**. Effect of USP8 inhibition on the level of intracellular bacteria in macrophages. **(A, B)** THP-1 macrophages were infected with GFP-expressing *Salmonella* at 24 hours after treatment with vehicle control, 2.5 μM DUB IN 2A, or 100 μg/mL ampicillin THP-1 nuclei and cytoplasm were stained using DPAI and HCS Cell Mask, respectively, and were imaged on the Cytation5 imaging platform **(A)**. Host cells, nuclei as well as GFP-tagged intracellular bacteria were visualized using fluorescent microscopy (Cytation 5), followed by counting of the GFP-tagged intracellular bacteria within the cell mask and host nuclei (n=3). Intracellular bacteria per 1000 cells is shown **(B)**. Significance was determined using a one-way ANOVA. Ampicillin was used as control, and vehicle control (VC) containing DMSO. The number of independent replicates for this experiment was 4 (n=4). **(C)** RAW264.7 macrophages were infected with GFP-*Salmonella* for 2 hours. Following this infection, cells were treated with 10, 5, 2.5, or 1.25 µM DUBs-IN2 USP8 inhibitor or 100 μg/mL ampicillin for 24 hours. Similar analysis of intracellular bacteria was done as in **(B)**. The number of independent replicates for this experiment was 3 (n=3). **(D, E).** THP-1 macrophages were infected with wild-type *Salmonella* Typhimurium for 1 hour followed by an additional hour incubation with 100 μg/mL of gentamicin to remove extracellular bacteria. Following this infection, cells were treated with 2.5 μM DUBs-IN2 USP8 inhibitor or 100 μg/mL ampicillin for either a 2- **(D)** or 24-hour **(E)** period. After these time points, the cells were lysed using 0.1% Triton-X100 with cell lysates being serially diluted to 10^-6^ and spot plated on LB Miller agar plates and incubated overnight at 37°C. CFU counts were taken for each treatment condition. The number of independent replicates for this experiment was 4 (n=4). P-values were indicated as follows: * p≤ 0.05; ** p ≤ 0.01; *** p≤ 0.001; **** p ≤ 0.0001.

To determine if inhibition of the USP8 protein leads to an overall decrease in bacterial survival in macrophages, we performed an infection of THP-1-derived macrophages using GFP-expressing *S.* Typhimurium and the treatment with USP8 selective inhibitor DUBs-IN2. In this assay, cells were infected (or not) with GFP-expressing *S.* Typhimurium, treated (or not) with the USP8 inhibitor, and then fixed. The host cell nuclei were stained for further counting of the cells. Moreover, the cell’s cytoplasm was stained to establish the cell perimeter. The images were analyzed in a high throughput manner, where bacteria were counted within the stained cytoplasm of the host cells and normalized to the nuclei count to determine the number of intracellular bacteria surviving the infection at 24 hours. Quantitative analysis indicated a significant reduction in intracellular *Salmonella* in THP-1 cells treated with 2.5 µM DUBs-IN2 compared to the vehicle control (**Figures 4A, B**). Additional infections performed in RAW 264.7 murine macrophages indicated similar results, where USP8 inhibitor DUBs-IN2 treatment was associated with a reduced number of *Salmonella* in the treated cells (**Figure 4C**; **Figure S3A**). In RAW 264.7 macrophages, a range of USP8 inhibitor concentrations (1.25-10 µM) was tested and, except 1.25 µM DUBs-IN2, all tested concentrations readily limited the number of intracellular bacteria (**Figure 4C**), which shows the dose-dependency of the effects of USP8 inhibitor on the intracellular number of bacteria. The DUBs-IN2 inhibitor also lowered the number of bacteria in HeLa cells (**Figure S3B**). However, Hela cells might represent a different mechanism of USP8-mediated reduction of intracellular bacteria compared to THP-1 and RAW 264.7 macrophage cell models since in epithelial cells, *Salmonella* invasion process depends on the bacterial secretory system proteins encoded within SPI-1 while the invasion of macrophages is controlled mainly by phagocytosis (Sansonetti, 2001). The survival of this bacterium within macrophages and epithelial cells also depends on different mechanisms (Knodler, 2015). We also verified that the GFP-*Salmonella* did not lose a significant amount of fluorescence by using gentamicin protection assay (**Figure S3C**). Finally, to verify that the GFP-*Salmonella* counts adequately reflect the reduction in the intracellular bacteria upon the USP8 inhibitor treatment, we performed a classical gentamicin protection assay, where the intracellular bacteria were enumerated by plating the THP-1 cell lysates on the agar plates to count the number of the colony forming units (CFUs). The CFUs of intracellular bacteria were enumerated after 2 hours (**Figure 4D**) and 24 hours post-infection (hpi) (**Figure 4E**), indicating that DUBs-IN2 USP8 inhibitor leads to a significant reduction of CFUs. In particular, at 24 hpi, there was a one-log of reduction observed in cells exposed to USP8 inhibitor DUBs-IN2 compared to vehicle control (**Figure 4E**). For ampicillin treatment, there was around a 2-log difference in the intracellular CFU number (**Figure 4E**), which is quite comparable to the results seen using microscopy counts (**Figure 4E**). Since the expression of SPI-2 appeared to be important in the regulation of USP8 expression (**Figure 2**), we also verified that the inhibition of USP8 in infections with *Salmonella* not expressing SseV that is therefore deficient in SPI-2, did not affect intracellular CFU number (**Figure S3D**). In summary, pharmacological inhibition of USP8 led to decreased bacterial survival of *Salmonella* in macrophages.

### 2.5 USP8 controls the autophagy flux during infection with *Salmonella*

**Figure 5:**
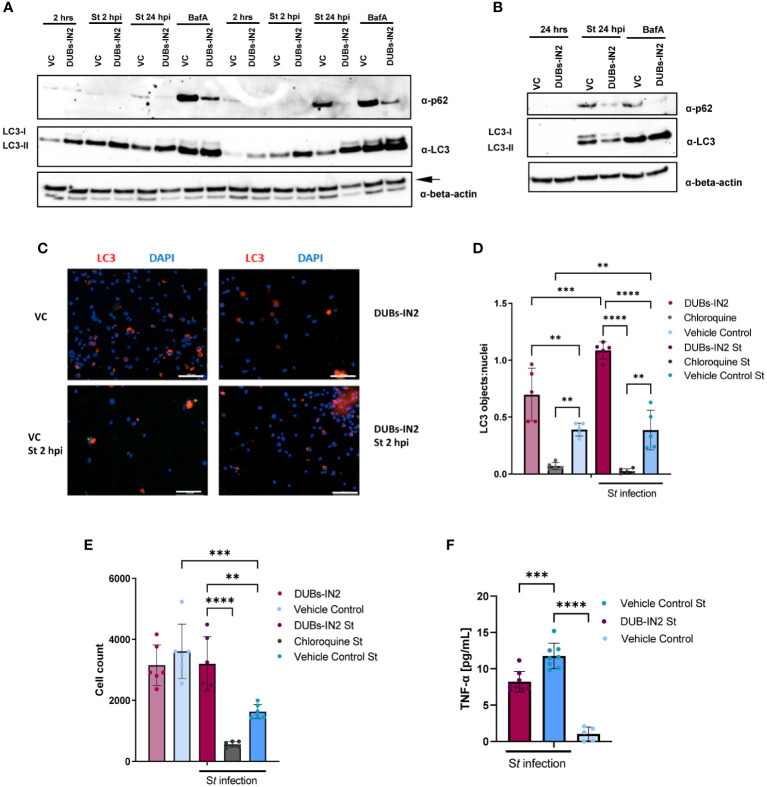
USP8 inhibition down-regulates p62 in human THP-1 macrophages during *S.* Typhimurium infection. **(A)**. THP-1-derived macrophages were treated with 2.5 µM DUBs-IN2 or vehicle control (VC) for 2 hours and then infected with *S.* Typhimurium (St) wild-type (MOI 50:1) for 2 hours or 24 hours (2 hpi or 24 hpi). The uninfected cells are indicated as “2 hrs”. Alternatively, cells were exposed to 100 nM bafilomycin A1 (BafA). Each sample was lysed, followed by western blotting to detect LC3 forms, p62 or β-actin (the latter one indicated with an arrow). The number of independent replicates for this experiment was 2 (n=2). **(B)**. THP-1- macrophages were treated with 2.5 µM DUBs-IN2 or vehicle control (VC) for 2 hours and then infected with *S.* Typhimurium (St) wild-type (MOI 50:1) for 24 hours (24 hpi) or left uninfected (24 hrs). Alternatively, cells were exposed to 100 nM bafilomycin A1 (BafA). Each sample was lysed, followed by western blotting to detect LC3 forms, p62 or β-actin. **(C-E)**. THP-1 macrophages seeded on 96-well plates were treated with 10 µM DUBs-IN2 or vehicle control (VC) for 2 hours, and then infected with *S.* Typhimurium wild-type (MOI 50:1) for 24 hours (24 hpi). Alternatively, cells were exposed to chloroquine [10 µM] for 2 hours before infection. Cells were stained with anti-LC3 antibody and DAPI. Six biological replicates were used per treatment (2x2 squares visualized under 10x objective). **(C)** DAPI-containing objects were automatically counted by using Gen5 imaging software. LC3-stained objects were also counted and normalized to the cell number (DAPI nuclei). A one-way ANOVA test was performed for statistical analysis of significance between the various treatments. The number of replicates for this experiment was 5 (n=5). **(D)** The cells from the experiment above **(A, B)** were counted as described above using the DAPI-stained nuclei. A one-way ANOVA test was performed for statistical analysis of significance between the various treatments **(E)** The number of replicates for this experiment was 5 (n=5). **(F)** Effect of USP8 inhibition on TNF-alpha secretion in infected cells. THP-1 macrophages were infected with *S.* Typhimurium for 1 hour followed by a 1-hour treatment with 100 μg/mL gentamicin to clear extracellular bacteria. Afterwards, the cells were incubated with 25 μg/mL of gentamicin and either a vehicle control or 2.5 μM of the DUBs-IN2 for 24-hour period, followed by measurement of TNF-alpha by ELISA. A one-way ANOVA test was performed for statistical analysis of significance between the various treatments. The number of replicates for this experiment was 8 (n=8). P-values were indicated as follows: * p≤ 0.05; ** p ≤ 0.01; *** p≤ 0.001; **** p ≤ 0.0001.

USP8 is involved in regulating protein trafficking to the lysosome (MacDonald et al., 2014) and the control of autophagic proteins (Gu et al., 2019). Hence, the effect of USP8 inhibition by DUBs-IN2 on autophagy during *Salmonella* infection and upon BafA treatment in THP-1 macrophages was tested by measuring p62 and LC3-II protein levels. USP8 inhibition was accompanied by a marked decrease in p62 adapter protein at 2 hpi and 24 hpi (**Figures 5A, B**). Moreover, USP8 also inhibited p62 expression upon BafA treatment (**Figure 5A**). Also, USP8 inhibition led to an increase in LC3-II during *Salmonella* infection but not upon BafA-mediated inhibition of autophagy (**Figure 5A**). Next, THP-1 cells were treated with DUBs-IN2 inhibitor and with *Salmonella* for 2 hours. Additional treatment with chloroquine was done as a control as chloroquine is a classic inhibitor of the autophagy process that should reduce the number of LC3 puncta. The antibody-stained LC3 puncta and DAPI-stained nuclei were visualized by microscopy (**Figure 5C**), and the number of LC3 objects was presented relative to the number of cells. USP8 inhibition was associated with an increased number of LC3 puncta as measured by fluorescent microscopy (**Figures 5D**, **S4**). While USP8 inhibition led to an increase in LC3 puncta in the absence of infection, the infection process further increased the increase in LC3 puncta. Some of these results showing the role of USP8 inhibition in regulating autophagy were replicated in HeLa cells (**Figure S5**), although it is essential to note that infection in epithelial HeLa cells might represent a separate mechanism compared to macrophages. Moreover, USP8 inhibition was associated with an increased survival o THP-1 macrophages upon *Salmonella* infection (**Figure 5E**). Finally, since *Salmonella* infection leads to an increase in pro-inflammatory TNF-α increase, we also tested whether this cytokine is affected by USP8 inhibition. There was a USP8-mediated reduction in pro-inflammatory TNF-α released from *Salmonella*-infected cells compared to vehicle control (**Figure 5F**).

In summary, USP8 inhibition has been shown to reduce the autophagy protein p62 during infection with *Salmonella*, which is correlated with increased autophagy flux.

## 3 Discussion

The rise of MDR *Salmonella* suggests that the alternative treatments and preventative means to tackle these infections are still limited (Coward et al., 2014). A better understanding of the innate responses to *Salmonella* infection will steam the discovery of new targets for therapeutic development. One of the critical processes by which intracellular *Salmonella* can be eliminated is xenophagy, a specialized form of autophagy geared toward eliminating intracellular bacteria (Wang et al., 2018). The fine-tuning of the autophagic signals and the presence of lysosomes and their functionality is critical, yet *Salmonella* is known to inhibit this process from limiting the xenophagy in macrophages (Thomas et al., 2012; Xu et al., 2019). Ubiquitination is a mechanism that plays a vital role in innate responses (Heaton et al., 2016; Lopez-Castejon and Edelmann, 2016) by controlling xenophagy (Alwan and van Leeuwen, 2007; Zhou et al., 2013; Scheidel et al., 2016; van Wijk et al., 2017) and other innate immune defenses [reviewed in (Lopez-Castejon and Edelmann, 2016; Wang et al., 2018)]. Deubiquitinases are a large family of enzymes involved in several aspects of the ubiquitination pathway [reviewed in (Komander, 2010)]. This study focused on identifying the functional inhibition of human deubiquitinases during the macrophage infection with *Salmonella* Typhimurium, which we compared to infection with another Gram-negative bacterium, *Yersinia enterocolitica*. Using chemical proteomics, where the functional labeling of active enzymes with the ubiquitin-specific active site-directed probe was coupled to quantitative mass spectrometry, we identified several deubiquitinases with activity and protein levels upregulated or downregulated by the host during these Gram-negative infections. Finally, a function of one of these deubiquitinases, USP8 was evaluated in the host response to *Salmonella* infection.

This study determined that USP8 is downregulated in macrophages during *Salmonella* infection but not during *Yersinia* infection, indicating that the regulation of this enzyme is not an unspecific response of the macrophages to Pathogen-associated molecular patterns (PAMPs) but a characteristic specific to this *Salmonella* infection model. Moreover, since inflammasome is activated in *Salmonella*-infected THP-1 macrophages, as we have previously shown (Sheppe et al., 2018), it would be interesting do repeat the same study but in cells where inflammasome has been activated in the absence of live bacteria. USP8 is an enzyme previously linked to the various processes involved in the endolysosomal pathway (Alwan and van Leeuwen, 2007; Zhou et al., 2013; MacDonald et al., 2014). USP8 depletion causes the accumulation of cation-independent M6P receptor (ci-M6PR) in the early endosome (MacDonald et al., 2014). These processes are linked with autophagy, an important pathway that can control intracellular *Salmonella* infection [reviewed in (Wang et al., 2018)]. *Salmonella* infects various cell types, including macrophages and epithelial cells, and its intracellular survival in all these cells depends on interference with the autophagy process (Alwan and van Leeuwen, 2007; Zhou et al., 2013; Scheidel et al., 2016; van Wijk et al., 2017). As a protective strategy, intracellular *Salmonella* survives in SCV, *Salmonella*-containing vacuole, to hide from the host responses leading to autophagy. If the SCV integrity is broken, cytosolic bacteria are ubiquitinated and targeted by OPTN, p62, NDP52 (van Wijk et al., 2017), then finally subjected to autophagy. In this process, galectin-8 is recruited immediately after sensing SCV damage, and it induces autophagy that is mediated by NDP52 (Thurston et al., 2012; Scheidel et al., 2016). The ubiquitin-dependent autophagy mechanism is activated, where *Salmonella* becomes ubiquitinated, followed by autophagy receptor recruitment, NDP52/p62/TBK1 (Rogov et al., 2013), and recognition of bacteria (Ivanov and Roy, 2009). Bacterial virulence factors inhibit autophagy, including SseL (Thomas et al., 2012), so there is a constant requirement for fine-tuning the ubiquitin processes related to autophagy. However, the deubiquitinases regulating these processes largely remain to be identified.

The roles of USP8 in xenophagy are not characterized, and there are conflicting results regarding the function of USP8 in autophagy. USP8 has been suggested to serve as a negative regulator of autophagy and deubiquitinate SQSTM1/p62 (Peng et al., 2019). The knockdown of USP8 promoted autophagic degradation of SQSTM1/p62 (p62), decreasing the p62 adaptor protein in HEK293T cells. In contrast, the overexpression of USP8 led to an increase in p62 in these cells (Peng et al., 2019). Interestingly, USP8 also enhances the interaction between EPG5 and LC3 in embryonic stem cells (EPCs), where EPG5 is a novel candidate for regulating autophagy in these cells (Gu et al., 2019); the knockdown of USP8 decreased the autophagy flux, while USP8 overexpression caused an increase in autophagy under normal and starvation conditions (Gu et al., 2019). Finally, depletion of USP8 leads to a steady-state redistribution of ci-M6PR from the Trans-Golgi Network to endosomal compartments, where USP8 might enhance the sorting of its substrates such as Cathepsin D. However, the USP8 substrates related to the regulation of autophagy remain unidentified (Berlin et al., 2010; MacDonald et al., 2014). In summary, USP8 appears to have conflicting roles in the process of autophagy, which indicates that it might be required for the proper functioning of this process in specific cells and under specific stimuli. To discover the USP8 role in infection, we used a chemical inhibition of USP8 by DUBs-IN-2 inhibitor (von Stockum et al., 2019). USP8 inhibition increased autophagy flux, as shown by the staining of LC3 puncta and clearance of p62. Hence, USP8 is proposed to suppress autophagic flux during infection with *Salmonella*, and its inhibition is associated with an induced clearance of bacteria. *Salmonella* has been shown to induce macrophage cell death by inducing autophagy (Hernandez et al., 2003). Furthermore, the presence of fully virulent bacteria was required for the downregulation of USP8. The presence of *Salmonella* Pathogenicity Island-2 (SPI-2) was required for infection-induced USP8 downregulation during infection. The SPI-2 genes are expressed when the bacterium is inside the phagosome of a macrophage, and SPI-2 effectors interfere with host signaling pathways to alter the phagosome into the *Salmonella*-containing vacuole (SCV) but also to acquire nutrients from the host cell (Liss et al., 2017).

In summary, this study discovered differential regulation of several deubiquitinating enzymes that control the ubiquitination process during infection with Gram-negative bacteria. We found that USP8 deubiquitinase is down-regulated during the *Salmonella* infection in human macrophages, which likely benefits the infected host since the pharmacological inhibition of this enzyme leads to the elimination of bacteria from the cells. USP8 inhibition was also shown to enhance autophagy, a host process involved in clearing intracellular bacteria. While several deubiquitinases have been identified as gatekeepers of the essential function of phagocytes (Edelmann et al., 2009; Basseres et al., 2010; Edelmann et al., 2010), the function of deubiquitinases in autophagy has not been well investigated. Since deubiquitinases are easily druggable enzymes, once we discover DUBs that are negative regulators of xenophagy or other innate responses, we can identify novel molecular mechanisms by which the DUBs downregulate the innate immune responses and find possible targets for host-directed therapies.

## 4 Methods

### 4.1 Cell culture

THP-1 monocytic cells (ATCC TIB-202; ATCC, USA) were cultured, as described previously [57 in RPMI 1640 (Gibco/Life Technologies, Inc., USA) supplemented with 10% fetal bovine serum (FBS), 2 mM GlutaMAX (Gibco/Life Technologies), and 100 μg/ml penicillin/streptomycin (Gibco/Life Technologies) in a humidified atmosphere of 5% CO2 at 37°C. For activation and differentiation of THP-1 cells into macrophages, 10 nM phorbol 12-myristate 13-acetate (PMA; Sigma-Aldrich, USA) was used, which was added for 48 hours. Human HeLa cells and RAW264.7 murine macrophages (ATCC# TIB-71, ATCC, USA) were cultured in DMEM media supplemented with 10% fetal bovine serum (FBS) and 100 μg/mL penicillin/streptomycin (Life Technologies Inc., USA).

### 4.2 Bacterial culture

The overnight cultures of *Salmonella enterica* serovar Typhimurium, strain 12023 or UK-1 χ3761 were cultivated at 37°C in lysogeny broth (LB) media overnight, shaking. Overnight bacterial cultures were diluted in fresh LB media in a 1:80 ratio to reach the optical density at 600 nm (OD600) of 0.05 and grown until OD600 was 0.50, followed by a wash by PBS and cells were subsequently resuspended in a cell culture medium. GFP-containing *Salmonella* UK-1 was generated by electroporation with pON::sfGFP plasmid as we did previously (Sheppe et al., 2021). Alternatively, we also used ΔssaV *S.* Typhimurium (Beuzón et al., 2000).

*Yersinia enterocolitica* 8081 (pYV) wild type (wt) was grown in LB media overnight (18 h) at 27°C. The culture was diluted in fresh LB to achieve a final optical density at 600 nm (OD_600_) of 0.05, and then incubated at 27°C for ∼2 h until the OD_600_ reached 0.25. The temperature was then changed to 37°, and bacteria were grown until the OD_600_ reached 0.5, followed by centrifugation at 5,000 × g. Bacteria were washed with PBS and resuspended in a cell culture medium.

### 4.3 Infection of cells for overexpression and inhibitor studies

From a THP-1 monocyte culture grown in suspension, a volume of media containing 6e^6^ cells was taken, and Phorbol-12-Myristate-13-Acetate (PMA) was added to a final concentration of 40 nM. The prepared cell suspension was transferred to a 96-well cell culture plate, so 6e^4^ monocytes were added to each well. The seeded THP-1 monocytes were incubated at 37°C in an atmosphere of 5% CO_2_ for 48 hours to ensure the differentiation of THP-1 monocytes into macrophages. Alternatively, HeLa cells and RAW 264.7 cells were prepared similarly, where cells were seeded 24 hours before infection. In some cases, HeLa cells were transfected with USP8-FLAG. Here, hUSP8 (UBPY) human wild-type was cloned into p3xFLAG-CMV7.1 using SalI-BamHi site, where N-terminal 3xFLAG epitope was in-frame with USP8. This hUSP8 cloned into p3xFLAG-CMV7.1 was transfected into the HeLa cells using Superfect reagent (Qiagen).

An overnight culture of GFP-expressing plasmid-transformed x3761 *S. enterica* serovar Typhimurium was prepared from individual colonies on plates added to LB Miller media containing 25 µg/mL of chloramphenicol as a selectivity marker for the plasmid. The bacterial culture was propagated under shaking at 200 rpm at 37°C overnight. On the next day, the subculture solution was prepared from the overnight culture at an OD_600_ = 0.050 in fresh LB Miller media containing no antibiotics and placed in an incubator for 3 hours at 200 rpm and 37°C. OD_600_ readings were taken at 1-hour intervals to monitor the progress of culture growth over the three hours. The OD_600_ was determined at the three-hour mark, and the CFU concentration was determined against a standardized OD_600_/CFU concentration. After determining the approximate bacteria concentration and acquiring an average cell count from the transformed THP-1-derived macrophages that were seeded two days prior, MOI 30 was prepared for the infection and applied to the cells in RPMI containing 10% FBS and no antibiotics (Incomplete RPMI) for one hour in an incubator at 37°C 5% CO_2_. After the one-hour incubation, the media was aspirated and replaced with incomplete RPMI containing 100 µg/mL gentamicin and incubated for an additional hour at 37°C in 5% CO_2-_DPBS containing Ca^2+^/Mg^2+^(Corning, Catalog: 21-030-CV). After the final 1-hour incubation, the media were aspirated and washed with 1 x mL D-PBS three times. Six replicates of the following treatments were used for the infection: DPBS with Ca^2+^/Mg^2+^. Next, the treatment compounds were prepared for 24-hour incubation in incomplete RPMI containing 25 ug/mL of gentamicin. Six replicates of the following treatments were used for the infection, including DUBs-IN-2 (HY-50737A, MedChem Express) USP8 Inhibitor at the indicated concentrations or 100 µg/mL of ampicillin which was used as a control treatment that should lead to a decrease in *Salmonella*. The treatments were applied to the cells and placed in a 37°C 5% CO_2_ incubator for 24 hours. RAW264.7 or HeLa cells were infected similarly, but incomplete DMEM media were used instead of RPMI media. At the 24-hour mark, the media were aspirated, and cells were washed twice with 1X DPBS containing Ca^2+^/Mg^2+,^ then fixed with 4% paraformaldehyde (PFA) for 10 minutes. The PFA was aspirated, and cells were washed twice with 1X DPBS with Ca^2+^/Mg^2+^ and then permeabilized with 0.1% Triton-X 100 for 15 minutes. Following permeabilization, the cells were aspirated and washed twice with 1X DPBS with Ca^2+^/Mg^2+^ and then stained with 100 nM of DAPI containing 2 µg/mL of HCS Cell Mask (Invitrogen, Catalog: H32721) solution for 30 minutes. After staining, the cells were washed using 1X DPBS with Ca^2+^/Mg^2+^ and left in 150 µL of 1X DPBS containing Ca^2+^/Mg^2+^ for imaging in the Cytation 5 (Biotek) system. The number of GFP-expressing bacteria and host cell nuclei were counted by the Gen5 Microplate Reader and Imager Software (Biotek) and ration of bacteria per host nuclei identified as we did previously (Sheppe et al., 2021).

### 4.4 Cytotoxicity/viability measurement

The cytotoxicity and viability of THP-1, HeLa, or RAW264.7 cells exposed to the DUBS-IN-2 inhibitor or ampicillin with infections performed as described above on 96-well plates were measured by using MultiTox-Fluor Multiplex Cytotoxicity kit (Promega Inc., Catalog: G9200).

### 4.5 Probe labeling and proteomics analysis

The THP-1 monocytes were grown in RPMI-1640 medium containing 10% heat-inactivated fetal bovine serum albumin and 1% combination penicillin/streptomycin antibiotics and differentiated to macrophages by 100 nM PMA for 48 hours on 10-cm dishes. The infections with *Salmonella* Typhimurium 12023 or *Y. enterocolitica* 8081 were performed as described above using mid-log bacterial cultures. However, after the initial 1-hour infection, media were removed, and the cells were incubated for 1 hour with incomplete media containing 100 µg/mL gentamicin, followed either for collection of the cells for the 2-hour infection period or by additional incubation (for 18-hour period) in RPMI media containing 20 µg/mL gentamicin. Cells were collected, washed in PBS, and frozen until further use. Cell pellets were resuspended in four pellet volumes of NP-40 Lysis buffer (150 mM NaCl, 20 mM MgCl2, 50 mM Tris [pH 7.5], 0.5% NP-40) containing 1 mM PMSF, 2 U/ml Turbo DNase, 20 U/ml RNase Cocktail (Ambion), and lysed for 30 minutes on ice. The samples were then centrifuged for 15 min at 21,000 x g, the supernatant was collected, and protein concentration was measured. The samples were then diluted 1:1 in 150 mM NaCl, 5 mM CaCl_2_, 50 mM Tris [pH 7.5], and DTT was added to a final concentration of 1 mM and incubated for 10 min on ice. After that, the HA-Ubiquitin-Vinyl Sulfone probe was used for *in vitro* labeling reaction and added to samples at 1:100 probe: protein ratio and incubated for 30 min at 37°C. The sample was then placed on ice and mixed with 100 µL washed and equilibrated EZview HA beads. The immunoprecipitation proceeded at 4°C for 2 hours, and samples were on a rotator. The resin was then washed four times with 800 µL of 150 mM NaCl and 50 mM Tris [pH 7.5]. Finally, proteins were eluted using 0.1% RapiGest SF (Waters), followed by boiling at 95°C for 5 minutes. Samples were then precipitated by 0.18 volumes of 100% TCA for 20 min on ice, spun for 30 min at 21,000 x g, and the precipitate was washed by 500 µL of -20°C acetone. The samples were spun again, acetone removed, and the precipitate was left to air dry for the tryptic digestion, which was performed as described before (Hui et al., 2021), and the samples were analyzed by LTQ Velos (Thermo Scientific), and data analysis was done by Proteome Discoverer and Scaffold) as done before (Hui et al., 2018). Tandem mass spectra were extracted by Proteome Discoverer. Charge state deconvolution and deisotoping were performed. All MS/MS samples were analyzed using Sequest (XCorr Only) (Thermo Fisher Scientific, San Jose, CA, USA; version 1.4.0.288) and X! Tandem (The GPM, thegpm.org; version CYCLONE (2010.12.01.1)). Sequest was set up to search the human Uniprot database (64,287 entries), assuming the digestion enzyme trypsin. Sequest and X! Tandem were searched with a fragment ion mass tolerance of 0.50 Da and a parent ion tolerance of 30 PPM. Carbamidomethyl of cysteine was specified in Sequest (XCorr Only) and X! Tandem as a fixed modification. Oxidation of methionine was specified in Sequest as a variable modification. Scaffold (version Scaffold 5.0.0, Proteome Software Inc., Portland, OR) was used to validate MS/MS-based peptide and protein identifications. Peptide identifications were accepted if they could be established at greater than 95.0% probability by the Peptide Prophet algorithm with Scaffold delta-mass correction. Protein identifications were accepted if they could be established at greater than 95.0% probability and contained at least two identified peptides. Protein probabilities were assigned by the Protein Prophet algorithm. Protein‐level false discovery rate (FDR) was a minimum 1%. Proteins that contained similar peptides and could not be differentiated based on MS/MS analysis alone were grouped to satisfy the principles of parsimony. The quantification was done using weighted spectral count where fold change was calculated between the proteins from infected samples compared to uninfected samples, and the samples were normalized to the overall spectral count. Fisher’s test was used to calculate the statistical significance of the findings. The data were deposited to Mendeley data (DOI: 10.17632/2hght7dmk5.1).

### 4.6 Western blot analysis

Cells were lysed as described in the section above, and protein concentration was quantified using a BCA protein assay kit (Thermo Fisher Scientific). Protein samples were separated by using 4 to 12% gradient SDS-PAGE and transferred onto a polyvinylidene difluoride membrane (Bio-Rad, USA), followed by blocking of the membrane with 5% nonfat milk in Tris-buffered saline (TBS) containing 0.1% Tween, and proteins of interest were detected by immunodetection with the appropriate antibodies and enhanced chemiluminescence. The following antibodies were used in this study: USP8 (#8728, Cell Signaling), OTUD6B (ab127714, Abcam), USP14 (#8159, Cell Signaling), USP15 (14354-1-AP, Proteintech), USP47 (sc-100633, Santa Cruz), UCH-L5 antibody (296, Santa Cruz Biotechnology, USA), β-actin (SC-47778, Santa Cruz), LC3A/B (#4108, Cell Signaling), goat anti-mouse antibody 31430 from Thermo Scientific, goat anti-rabbit antibody 31460 from Thermo Scientific, and donkey anti-goat antibody HAF017 from R&D Systems. All secondary antibodies were horseradish peroxidase-conjugated and prepared fresh for each Western blot. Primary antibodies were stored in 0.5% nonfat milk in TBS containing 0.1% Tween and 0.02% sodium azide at 4°C.

### 4.7 Fluorescent microscopy

For LC3 staining, THP-1 cells were grown on 96-well plates, treated with DUBs-IN2 inhibitor, and/or infected with *S.* Typhimurium for 24 hrs. Alternatively, cells were also exposed to 10 µM chloroquine. After the infection was complete, the growth media were removed and washed twice with PBS. Next, cells were fixed by incubation with 30 µL/well of 4% paraformaldehyde (PFA) for 30 minutes at room temperature. PFA was removed, and wells were washed three times with PBS. Cells were then permeabilized with 0.1% TritonX-100 for 5 minutes at room temperature, followed by three washed with PBS. Cells were blocked with 50 µL of 5% goat serum for 60 minutes at room temperature, followed by two washed with PBS. Next, the primary antibody (Novus Biologicals rabbit anti-LC3 primary antibody; NB100- 2220) was diluted in 1% goat serum, and cells were incubated for 1 hour at room temperature. Thereafter, cells were washed three times with PBS, and the secondary anti-rabbit antibody was diluted in 1% goat serum in PBS, followed by one-hour incubation. Cells were washed twice with PBS and stained with DAPI for 7 minutes at room temperature in the dark, followed by two washes with PBS. The cells were then imaged using Cytation 5 (Biotek), equipped with a fluorescence microscope. The LC3 objects and nuclei were counted using the Gen5 Software (Biotek).

### 4.8 Gentamicin protection assay

For the inhibitor studies, PMA-differentiated THP-1 cells were infected with wild-type *Salmonella* Typhimurium for 1 hour followed by a 1-hour treatment with 100 μg/mL gentamicin to clear extracellular bacteria. In some experiments, Typhimurium 12023 (wt) or ΔssaV mutant were used. The cells were incubated with 25 μg/mL of gentamicin and either a vehicle control, a 100 μg/mL ampicillin control, or 2.5 μM of the DUBs-IN2 inhibitor treatment for either a 2- or 24-hour period. After these time points, the cells were washed twice with 1x DPBS and lysed with 0.1% Triton-X100 at 37°C for 10 minutes. Cell lysates were serially diluted to 10-6 in 1x PBS, and 10 μL of each of the diluted cell lysates were spot plated on LB Miller agar plates and incubated overnight. CFU/mL calculations were determined based on the resulting colony growth, and data were analyzed by GraphPad Prism.

### 4.9 TNF-alpha ELISA

Cells were infected with wild-type *Salmonella* as described above, and media were collected 24-hour post-infection and analyzed by the anti-TNF-alpha ELISA (Invitrogen). The data were analyzed by GraphPad Prism, followed where One-way ANOVA test was used to establish significance of the data.

## Data availability statement

The datasets presented in this study can be found in online repositories. The data will be available here: Hercik, Kamil; Edelmann, Mariola (2023), “Deubiquitinases in macrophages infected with Salmonella enterica or Yersinia enterocolitica”, Mendeley Data, V1, doi: 10.17632/2hght7dmk5.1.

## Author contributions

ME, KH, and GM contributed to the conception and design of the study. JS, MO, GM, and KH helped with the data acquisition and experimentation. JS and ME performed the statistical analysis. ME and JS wrote the first draft of the manuscript. ME, JS, KH, MO, and GM wrote sections of the manuscript. All authors contributed to the article and approved the submitted version.
