# Supplementary figures and images for: USP8 inhibition regulates autophagy flux and controls Salmonella infection
Source: Front Cell Infect Microbiol. 2023 Mar 21;13:1070271. doi: 10.3389/fcimb.2023.1070271 (PMC10072284; doi:10.3389/fcimb.2023.1070271)

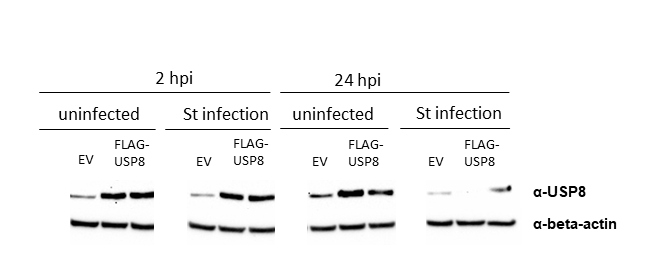

Supplement: Supplementary file 1 [file Image_1.tif]

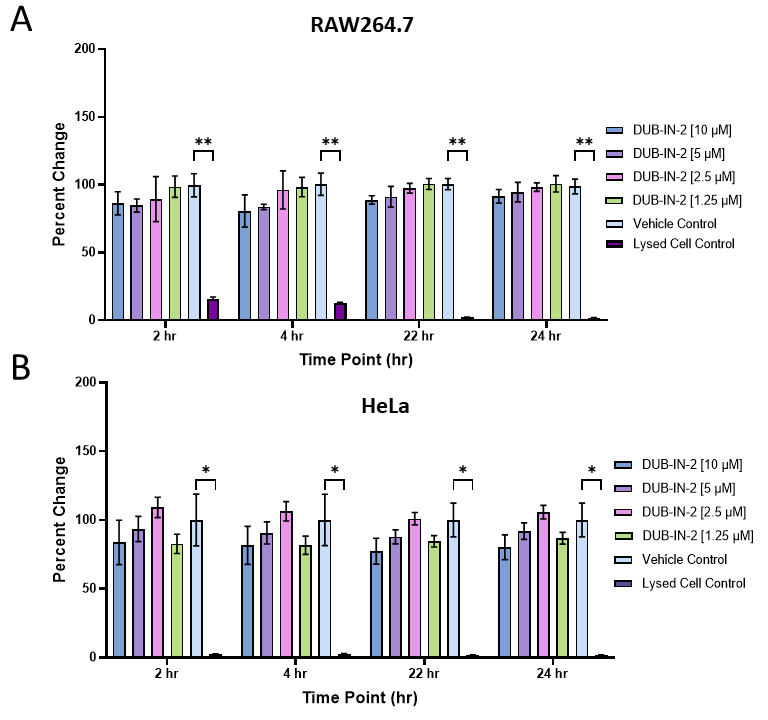

Supplement: Supplementary file 2 [file Image_2.tif]

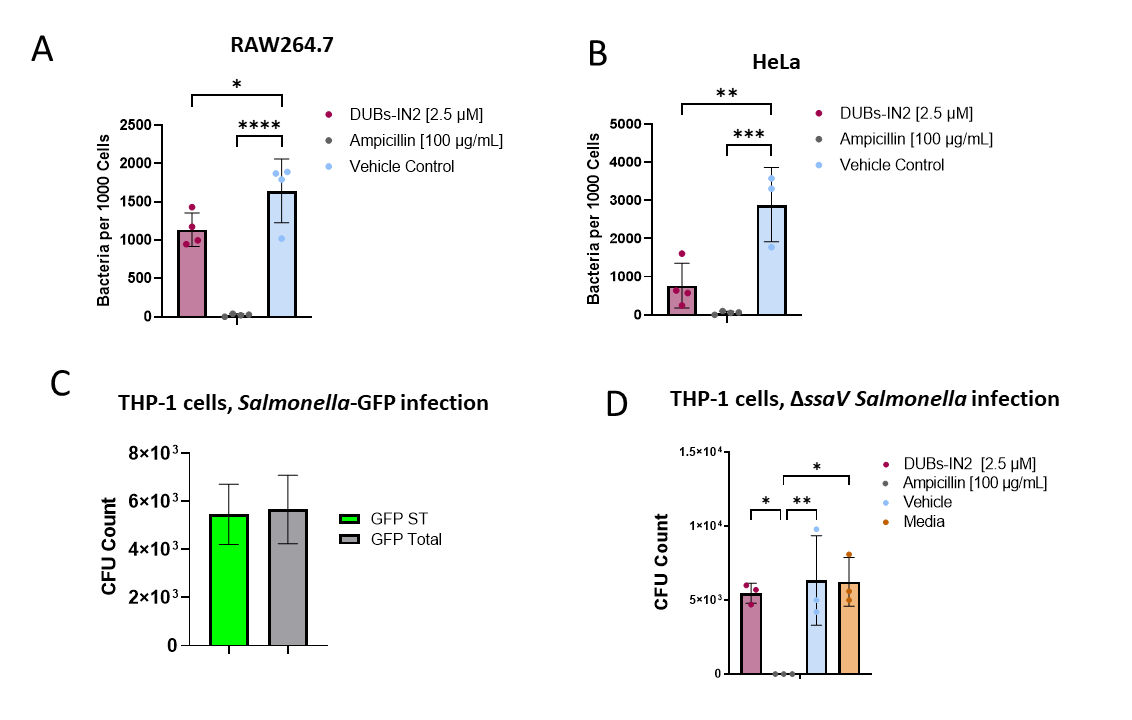

Supplement: Supplementary file 3 [file Image_3.tif]

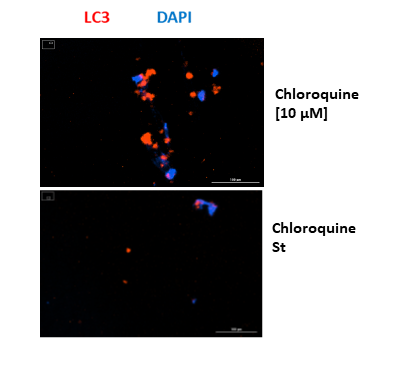

Supplement: Supplementary file 4 [file Image_4.tif]

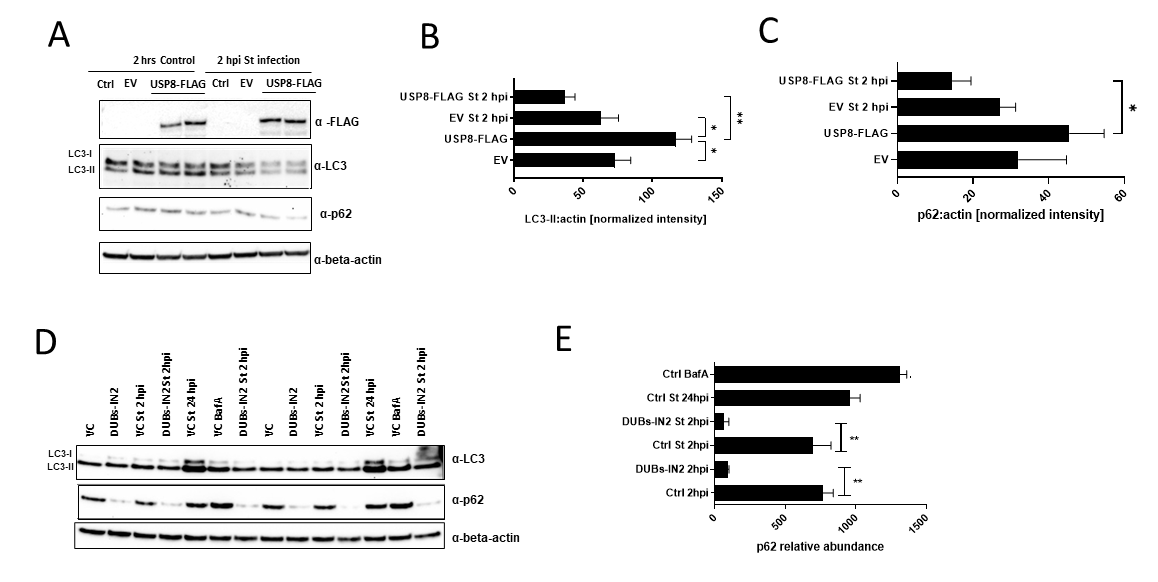

Supplement: Supplementary file 5 [file Image_5.tif]
